# CDK1 inhibition reduces osteogenesis in endothelial cells in vascular calcification

**DOI:** 10.1172/jci.insight.176065

**Published:** 2024-01-23

**Authors:** Yan Zhao, Yang Yang, Xiuju Wu, Li Zhang, Xinjiang Cai, Jaden Ji, Sydney Chen, Abigail Vera, Kristina I. Boström, Yucheng Yao

**Affiliations:** 1 Division of Cardiology, David Geffen School of Medicine at UCLA, Los Angeles, California, USA.; 2 The Molecular Biology Institute at UCLA, Los Angeles, California, USA.

**Keywords:** Cell biology, Vascular biology, Cardiovascular disease

## Abstract

Vascular calcification is a severe complication of cardiovascular diseases. Previous studies demonstrated that endothelial lineage cells transitioned into osteoblast-like cells and contributed to vascular calcification. Here, we found that inhibition of cyclin-dependent kinase (CDK) prevented endothelial lineage cells from transitioning to osteoblast-like cells and reduced vascular calcification. We identified a robust induction of CDK1 in endothelial cells (ECs) in calcified arteries and showed that EC–specific gene deletion of CDK1 decreased the calcification. We found that limiting CDK1 induced E-twenty-six specific sequence variant 2 (ETV2), which was responsible for blocking endothelial lineage cells from undergoing osteoblast differentiation. We also found that inhibition of CDK1 reduced vascular calcification in a diabetic mouse model. Together, the results highlight the importance of CDK1 suppression and suggest CDK1 inhibition as a potential option for treating vascular calcification.

## Abstract 2

CDK1 inhibition prevents vascular calcification through induction of E-twenty-six specific sequence variant 2.

## Introduction

Vascular calcification is a severe complication of cardiovascular disease that results in an increase in morbidity and mortality (1). It worsens atherosclerosis and is associated with an increased risk of congestive heart failure, myocardial infarction, and systemic hypertension (2–4). It is also a strong predictor of cardiovascular disease, stroke, and diabetic amputation (5). Furthermore, vascular calcification is a substantial problem in chronic kidney disease, and commonly complicates vascular procedures and surgeries (6, 7). Vascular calcification is known to be an active process involving ectopic bone formation, in which osteogenic differentiation occurs in cells transitioned from other lineages, including endothelial cells (ECs) (8–10).

The ill-fated vascular ECs can become a source of osteoprogenitors through endothelial-mesenchymal transitions (EndMTs) and contribute to calcification (11–17). In this process, ECs gain plasticity and differentiate into osteoblast-like cells. This dramatic switch of cell fate has been shown to contribute to vascular calcification in diabetes mellitus, where hyperglycemia induces bone morphogenetic proteins (BMPs) and drives ECs to transition into osteoblast-like cells, thereby causing arterial calcification (11–21). Osteoblast-like cells with endothelial characteristics can be found in calcified lesions of diabetic aortic tissue (14–16, 18, 20). Although previous studies showed that EC-derived osteogenesis plays an important role in vascular calcification, it remains unclear how to control the EC fate in diseased microenvironments in order to prevent EC-derived osteogenesis.

The regulators of EC fate during the developmental process and vascular plexus remodeling have been closely studied in recent years (22, 23). These studies show that the distribution of essential growth factors balance the activity of critical transcription factors to direct EC differentiation and proliferation (22, 23). Interestingly, a previous study also suggested that EC fate is affected by the dynamic regulation of the cell cycle through BMP and TGF-β signaling (24), suggesting that the regulators of the cell cycle may be involved in the shift in EC fate. Cyclin-dependent kinases (CDKs) are important components of cell cycle regulation (25). Here, we investigated the role of CDKs in vascular calcification. We discovered that CDK1 inhibition induced E-twenty-six specific sequence variant 2 (ETV2) to prevent the differentiation of EC-derived osteoblast-like cells and reduce arterial calcification in matrix Gla protein–deficient (*Mgp^–/–^*) mice, a model of vascular calcification (14–16, 26) and in diabetic *Ins2^Akita/+^* mice, a model of vascular calcification in diabetes (15–18).

## Results

### CDK inhibitor AT7519 prevents endothelial lineage cells from undergoing osteogenic differentiation and reduces arterial calcification in Mgp^–/–^ mice.

**Figure 1:**
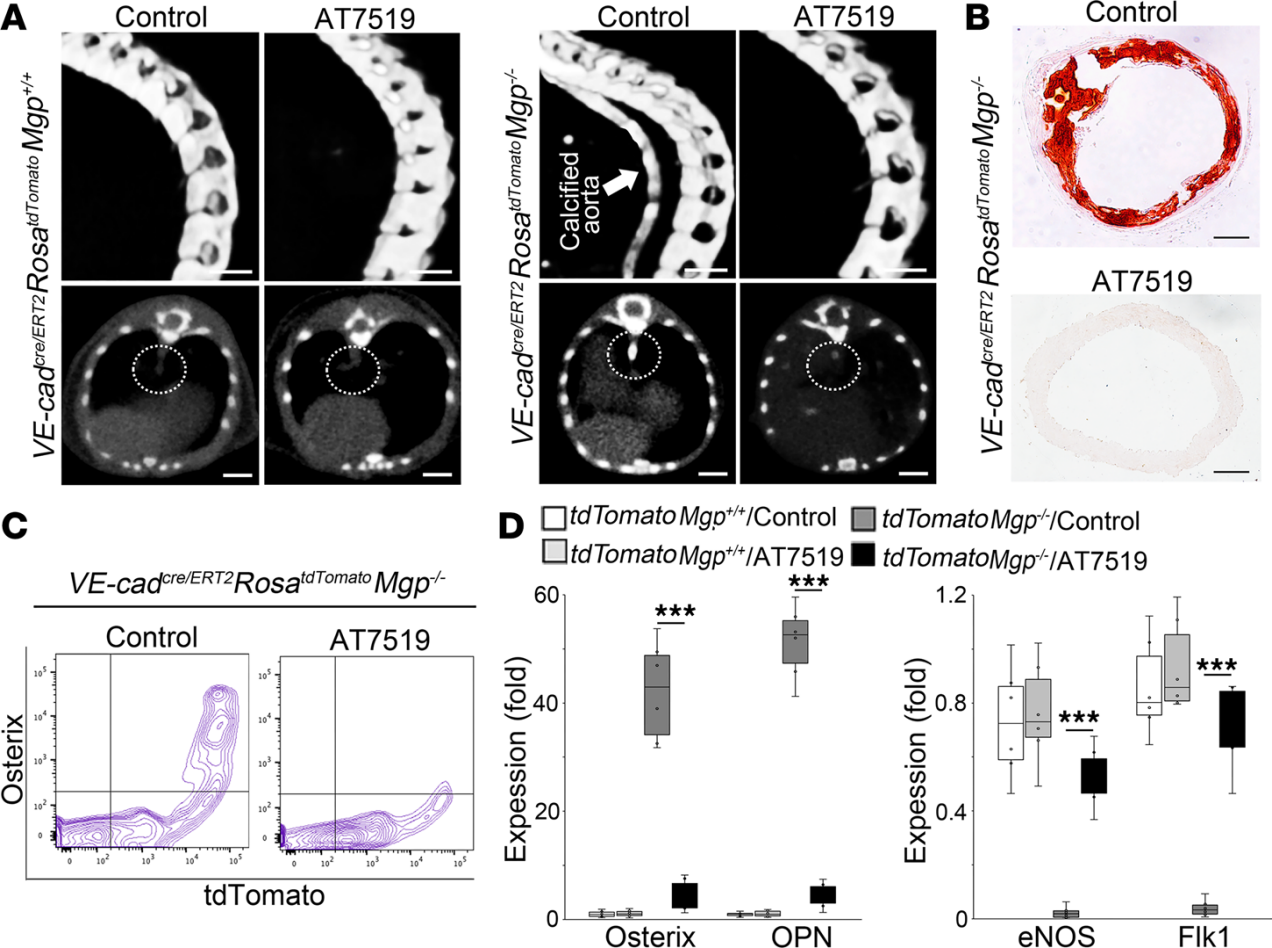
CDK inhibitor AT7519 prevents endothelial lineage cells from osteogenic differentiation and reduces arterial calcification in *Mgp^–/–^* mice. (**A**) Micro-CT imaging of *VE-cadherin^cre/ERT2^* *Rosa^tdTomato^* *Mgp^–/–^* mice and *VE-cadherin^cre/ERT2^* *Rosa^tdTomato^* mice after the administration of AT7519 (*n* = 6). Saline was used as control. Arrow and circles highlight the aortic calcification. Scale bars: 5 mm. (**B**) Alizarin red staining of descending aortas of *VE-cadherin^cre/ERT2^* *Rosa^tdTomato^* *Mgp^–/–^* mice after the administration of AT7519 (*n* = 6). Scale bars: 100 μm. (**C**) FACS analysis of aortic cells isolated from *VE-cadherin^cre/ERT2^* *Rosa^tdTomato^* *Mgp^–/–^* mice after the administration of AT7519 (*n* = 6). (**D**) Expression of the osteogenic markers osterix and osteopontin (OPN) and the endothelial markers eNOS and Flk1 in tdTomato^+^ aortic cells isolated from *VE-cadherin^cre/ERT2^* *Rosa^tdTomato^* *Mgp^–/–^* mice and *VE-cadherin^cre/ERT2^* *Rosa^tdTomato^* mice after the administration of AT7519, shown by real-time PCR (*n* = 6). Data in **D** were analyzed for statistical significance by 1-way ANOVA with Tukey’s multiple-comparison test. The bounds of the boxes are upper and lower quartiles with data points, the line in the box is the median, and whiskers are maximal and minimal values. ***P < 0.0001.

To examine endothelial lineage cells in arterial calcification, we created *VE-cadherin^cre/ERT2^* *Rosa^tdTomato^* *Mgp^–/–^* mice, and administered tamoxifen to the mice at 1 week of age. *VE-cadherin^cre/ERT2^* *Rosa^tdTomato^* mice were used as controls. At 4 weeks of age, we confirmed the severe aortic calcification in *VE-cadherin^cre/ERT2^* *Rosa^tdTomato^* *Mgp^–/–^* mice using micro-CT and Alizarin red staining (Figure 1, A and B). FACS identified a cell population coexpressing tdTomato and osterix in aortic cells from *VE-cadherin^cre/ERT2^* *Rosa^tdTomato^* *Mgp^–/–^* mice (Figure 1C). We isolated tdTomato^+^ aortic cells from *VE-cadherin^cre/ERT2^* *Rosa^tdTomato^* *Mgp^–/–^* mice; real-time PCR showed induction of the osteogenic markers osterix and osteopontin, with a reduction in the endothelial markers eNOS and Flk1 (Figure 1D), supporting the notion that endothelial lineage cells transitioned into osteoblast-like cells in aortic calcification.

Interestingly, after we injected the *VE-cadherin^cre/ERT2^* *Rosa^tdTomato^* *Mgp^–/–^* mice with tamoxifen for 5 days followed by the injection of CDK inhibitor AT7519 (5 μg/kg, daily) for 2 weeks, micro-CT imaging and Alizarin red staining showed a dramatic reduction in aortic calcification (Figure 1, A and B). FACS also uncovered a reduction in cells that coexpressed tdTomato and osterix in the AT7519-treated group (Figure 1C). We isolated the tdTomato^+^ aortic cells and showed by real-time PCR that AT7519 prevented the induction of osteogenic markers as well as the reduction of endothelial markers in the cells from *VE-cadherin^cre/ERT2^* *Rosa^tdTomato^* *Mgp^–/–^* mice (Figure 1D). These results suggested that CDK inhibition prevented endothelial lineage cells from undergoing osteogenic differentiation and reduced vascular calcification.

**Figure 2:**
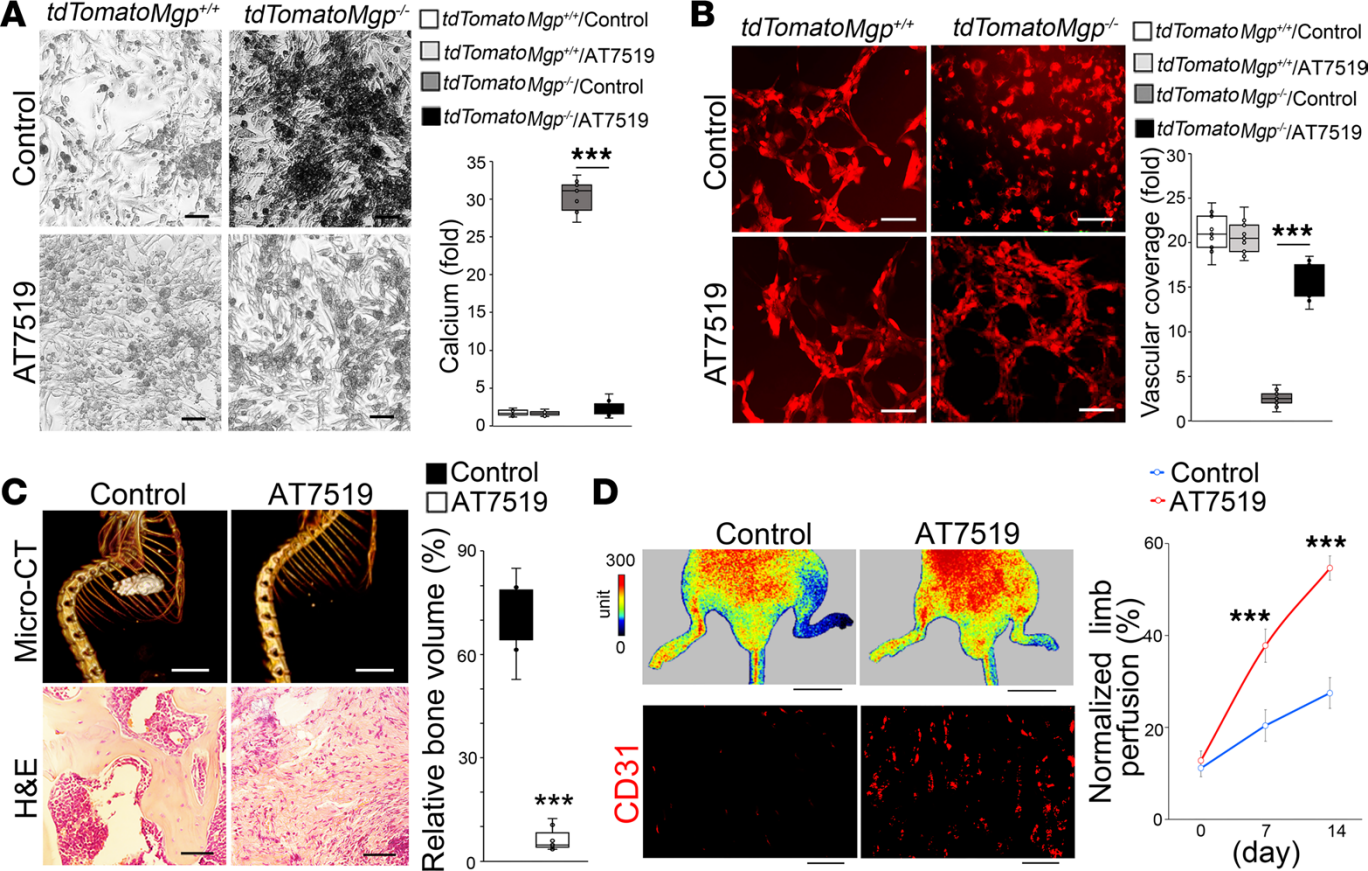
AT7519 prevents endothelial lineage cells from acquiring osteogenic capacity. (**A**) Von Kossa staining and total calcium quantitation in osteogenesis assays using tdTomato^+^ aortic cells isolated from *VE-cadherin^CreERT2^* *Rosa^tdTomato^* *Mgp^–/–^* mice treated with AT7519 (*n* = 9). *VE-cadherin^CreERT2^* *Rosa^tdTomato^* mice were used as controls. Scale bars: 50 μm. (**B**) Tube formation assays using tdTomato^+^ aortic cells isolated from *VE-cadherin^CreERT2^* *Rosa^tdTomato^* *Mgp^–/–^* mice treated with AT7519 (*n* = 9). *VE-cadherin^CreERT2^* *Rosa^tdTomato^* mice were used as controls. Red, tdTomato. Scale bars: 50 μm. (**C**) Micro-CT images of ectopic bone formation with analysis of relative volume of bone formation and H&E staining after transplantation of tdTomato^+^ aortic cells isolated from *VE-cadherin^CreERT2^* *Rosa^tdTomato^* *Mgp^–/–^* mice treated with AT7519 (*n* = 7). Saline was used as control. Scale bars: 5 mm (top) and 50 μm (bottom). (**D**) Laser Doppler perfusion images and immunostaining using anti-CD31 antibodies at ischemic sites after the transplantation of tdTomato^+^ aortic cells isolated from *VE-cadherin^CreERT2^* *Rosa^tdTomato^* *Mgp^–/–^* mice treated with AT7519 (*n* = 8). Saline was used as control. Scale bars: 10 mm (top) and 50 μm (bottom). Data were analyzed for statistical significance by 1-way ANOVA with Tukey’s multiple-comparison test (**A**, **B**, and **D**) or unpaired, 2-tailed Student’s *t* test (**C**). In **A**–**C**, the bounds of the boxes are upper and lower quartiles with data points, the line in the box is the median, and whiskers are maximal and minimal values. Data in **D** are presented as mean ± SD. ***P < 0.0001.

To further test this, we isolated tdTomato^+^ aortic cells from *VE-cadherin^cre/ERT2^* *Rosa^tdTomato^* *Mgp^–/–^* mice with or without AT7519 treatment. We cultured the cells in osteogenic induction media for 2 weeks. Von Kossa staining showed a strong mineralization in cells of the group without AT7519 treatment but decreased mineralization in the group treated with AT7519 (Figure 2A). Determination of total calcium validated the results of mineral deposition in the cells (Figure 2A). In parallel, we examined the capacity of the tdTomato^+^ aortic cells for tube formation. The results showed stronger tube formation by the tdTomato^+^ cells from the group treated with AT7519 than the cells from the group without AT7519 treatment (Figure 2B). These in vitro results suggested that CDK inhibition promoted the characteristics of *Mgp^–/–^* aortic endothelial lineage cells.

To assess potential changes in tdTomato^+^ cells in vivo, we first performed transplantation experiments to examine the osteogenic capacity using an ectopic bone formation assay (27). We isolated tdTomato^+^ aortic cells from *VE-cadherin^cre/ERT2^* *Rosa^tdTomato^* *Mgp^–/–^* mice with or without AT7519 treatment and transplanted the cells into nude mice, as previously described (28). Two weeks after transplantation, micro-CT imaging and H&E staining showed reduced amounts of ectopic bone, with less trabecular formation and bone volume in the implants with the cells from mice with AT7519 treatment compared with those without AT7519 treatment (Figure 2C), suggesting that AT7519 prevented *Mgp^–/–^* endothelial lineage cells from acquiring osteogenic capacity.

We also evaluated the endothelial capacity of the tdTomato^+^ aortic cells during vascular repair in the hindlimb ischemia model. After ligation of the proximal and distal femoral artery, tdTomato^+^ aortic cells were transplanted into the injury sites. Laser Doppler perfusion imaging demonstrated significantly higher limb blood flow in the mice transplanted with the cells from mice with AT7519 treatment than controls (Figure 2D), suggesting that AT7519 treatment promoted the capacity of *Mgp^–/–^* endothelial lineage cells for vascular repair.

### Limiting CDK1 prevents osteogenic differentiation in MGP-depleted ECs.

**Figure 3:**
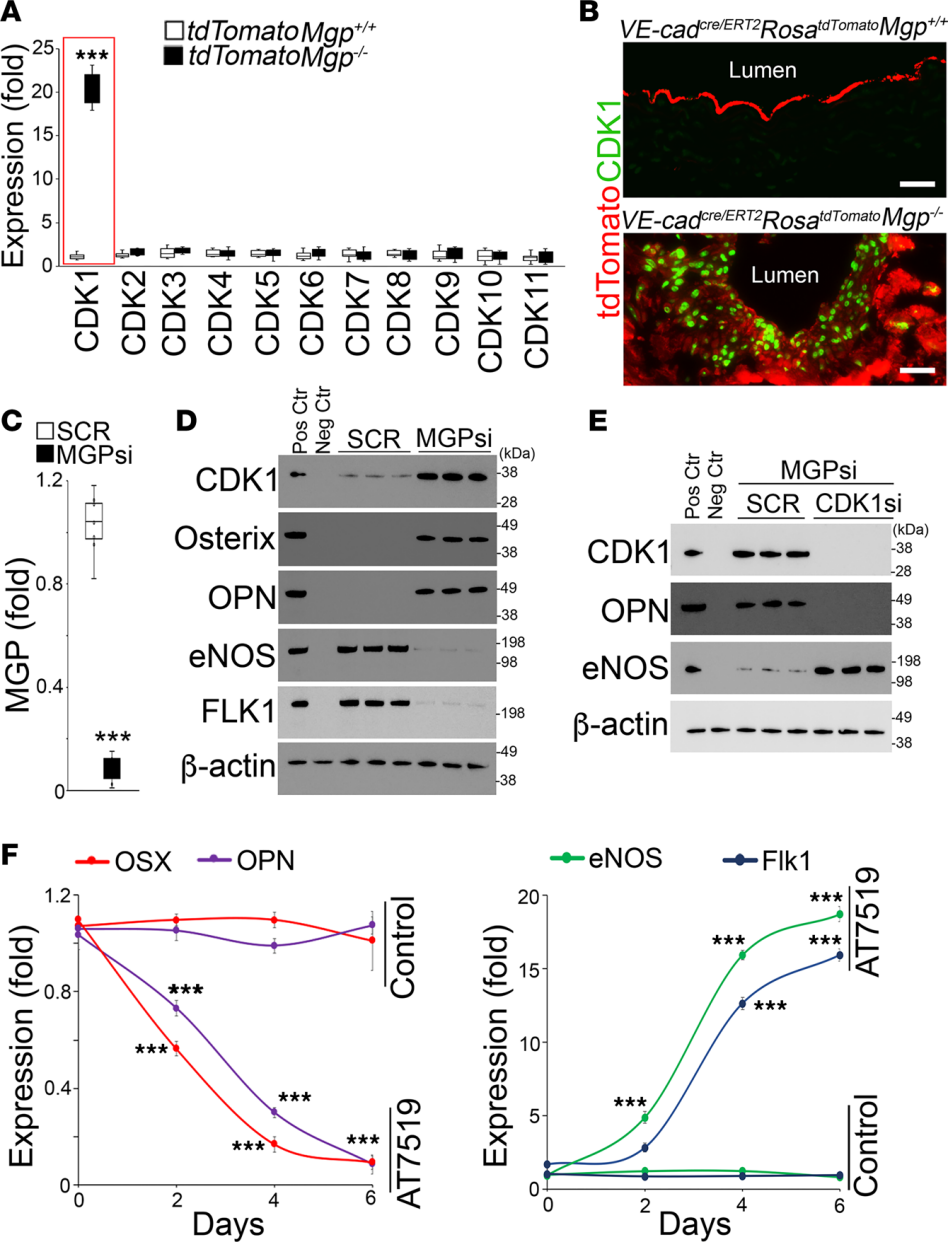
Inhibition of CDK1 prevents osteogenic differentiation in MGP-depleted ECs. (**A**) Expression of CDKs in tdTomato^+^ aortic cells isolated from *VE-cadherin^cre/ERT2^* *Rosa^tdTomato^* *Mgp^–/–^* mice and *VE-cadherin^cre/ERT2^* *Rosa^tdTomato^* mice, as determined by real-time PCR (*n* = 6). (**B**) Immunostaining of CDK1 in the aortic tissues of *VE-cadherin^cre/ERT2^* *Rosa^tdTomato^* *Mgp^–/–^* mice and *VE-cadherin^cre/ERT2^* *Rosa^tdTomato^* mice (*n* = 6). Scale bars: 50 μm. (**C**) Expression of MGP in HAECs after transfection of MGP siRNAs (*n* = 8). SCR, scramble siRNA. (**D**) Immunoblotting of CDK1, osteogenic, and endothelial markers in HAECs after transfection of MGP siRNAs and treatment with osteogenic induction media. Positive control (Pos Ctr) for CDK1 blotting: MCF-7 cells as verified by Thermo Fisher Scientific. Negative control (Neg Ctr) for CDK1 blotting: MCF-7 cells transfected with CDK1 siRNA. Positive control for osterix and osteopontin (OPN) blotting: MC3T3 osteoblast cells. Negative control for osterix and OPN blotting: HAECs. Positive control for eNOS and FLK1 blotting: HAECs. Negative control for eNOS and FLK1 blotting: MC3T3 osteoblast cells. Each lane represents an independent experimental group. (**E**) Immunoblotting of CDK1, OPN, and eNOS in HAECS after transfection of MGP siRNA in combination of with CDK1 siRNA and treatment with osteogenic induction media. Positive and negative controls are the same as in **D**. Each lane represents an independent experimental group. (**F**) Time-course expression of osteogenic and endothelial markers in HAECs after transfection of MGP siRNA and treatment with AT7519 or saline control (*n* = 3). Data were analyzed for statistical significance by unpaired, 2-tailed Student’s *t* test (**A** and **C**) or 1-way ANOVA with Tukey’s multiple-comparison test (**F**). In **A** and **C**, the bounds of the boxes are upper and lower quartiles with data points, the line in the box is the median, and whiskers are maximal and minimal values. Data in **F** are presented as mean ± SD. ***P < 0.0001.

To determine which CDK might be involved in the osteogenic transition of endothelial lineage cells, we examined the expression of the CDK family in the tdTomato^+^ aortic cells from *VE-cadherin^cre/ERT2^* *Rosa^tdTomato^* *Mgp^–/–^* mice. Real-time PCR showed a significant induction of CDK1 (Figure 3A), and immunostaining revealed strong CDK1 staining in the nuclei of the tdTomato^+^ aortic cells (Figure 3B). To test the role of endothelial CDK1 in calcification, we utilized human aortic ECs (HAECs), as previously described (16). We depleted MGP in HAECs using specific siRNA for MGP (Figure 3C), and cultured them in osteogenic induction media for 2 weeks. Immunoblotting showed an induction of CDK1, osterix, and osteopontin, but a reduction in eNOS and FLK1 in the MGP-depleted HAECs (Figure 3D). Similarly, we depleted both MGP and CDK1 in the HAECs. Immunoblotting showed that CDK1 depletion abolished the induction of osteopontin but enhanced eNOS expression in MGP-depleted HAECs (Figure 3E). We treated the MGP-depleted HAECs with 5 μM AT7519 for 1 week. A time course using real-time PCR showed that AT7519 reduced the expression of osterix and osteopontin but increased the expression of eNOS and Flk1 (Figure 3F). The results suggested that limiting CDK1 prevented endothelial lineage cells from undergoing osteogenic differentiation.

**Figure 4:**
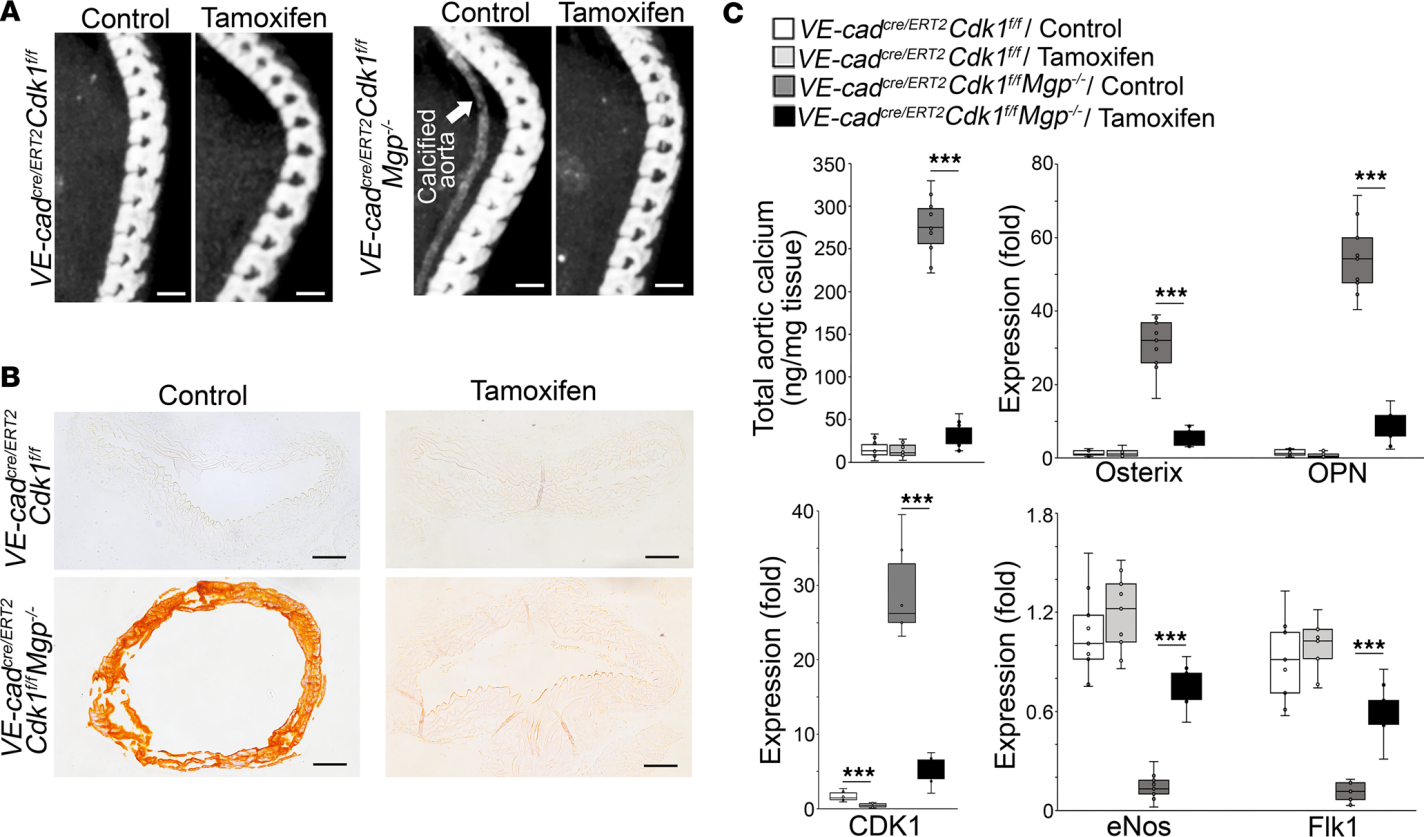
Endothelial deletion of CDK1 prevents aortic endothelial lineage cells from undergoing osteogenic differentiation and reduces aortic calcification. (**A**) Micro-CT imaging of *VE-cadherin^cre/ERT2^* *Cdk1^fl/fl^* *Mgp^–/–^* mice and *VE-cadherin^cre/ERT2^* *Cdk1^fl/fl^* mice after tamoxifen administration (*n* = 10). Saline was used as control. Arrow and circles highlight the calcification. Scale bars: 5 mm. (**B**) Alizarin red staining of descending aortas of *VE-cadherin^cre/ERT2^* *Cdk1^fl/fl^* *Mgp^–/–^* mice and *VE-cadherin^cre/ERT2^* *Cdk1^fl/fl^* mice after tamoxifen administration (*n* = 6). Scale bars: 100 μm. (**C**) Total aortic calcium and expression of Cdk1 and osteogenic and endothelial markers in CD31^+^CD45^–^ aortic cells isolated from *VE-cadherin^cre/ERT2^* *Cdk1^fl/fl^* *Mgp^–/–^* mice and *VE-cadherin^cre/ERT2^* *Cdk1^fl/fl^* mice after tamoxifen administration (*n* = 6). Data in **C** were analyzed for statistical significance by 1-way ANOVA with Tukey’s multiple-comparison test. The bounds of the boxes are upper and lower quartiles with data points, the line in the box is the median, and whiskers are maximal and minimal values. ***P < 0.0001.

To test this in vivo, we generated *VE-cadherin^cre/ERT2^* *Cdk1^fl/fl^* *Mgp^–/–^* mice, in which tamoxifen would be expected to reduce endothelial CDK1. We administered tamoxifen to the mice at 1 week of age. At 4 weeks of age, micro-CT imaging and Alizarin red showed a decrease in aortic calcification in the *VE-cadherin^cre/ERT2^* *Cdk1^fl/fl^* *Mgp^–/–^* mice compared with controls without tamoxifen treatment (Figure 4, A and B). Determination of the total aortic calcium confirmed the decrease in aortic mineral deposition in the tamoxifen-treated *VE-cadherin^cre/ERT2^* *Cdk1^fl/fl^* *Mgp^–/–^* mice (Figure 4C). Real-time PCR revealed a reduction in aortic CDK1, osterix, and osteopontin, with an increase in eNOS and Flk1 (Figure 4C). The results suggested that EC–specific deletion of CDK1 prevented osteogenic differentiation in endothelial lineage cells and reduced calcification.

### Limiting CDK1 upregulated ETV2 and prevented osteogenic differentiation in endothelial lineage cells.

**Figure 5:**
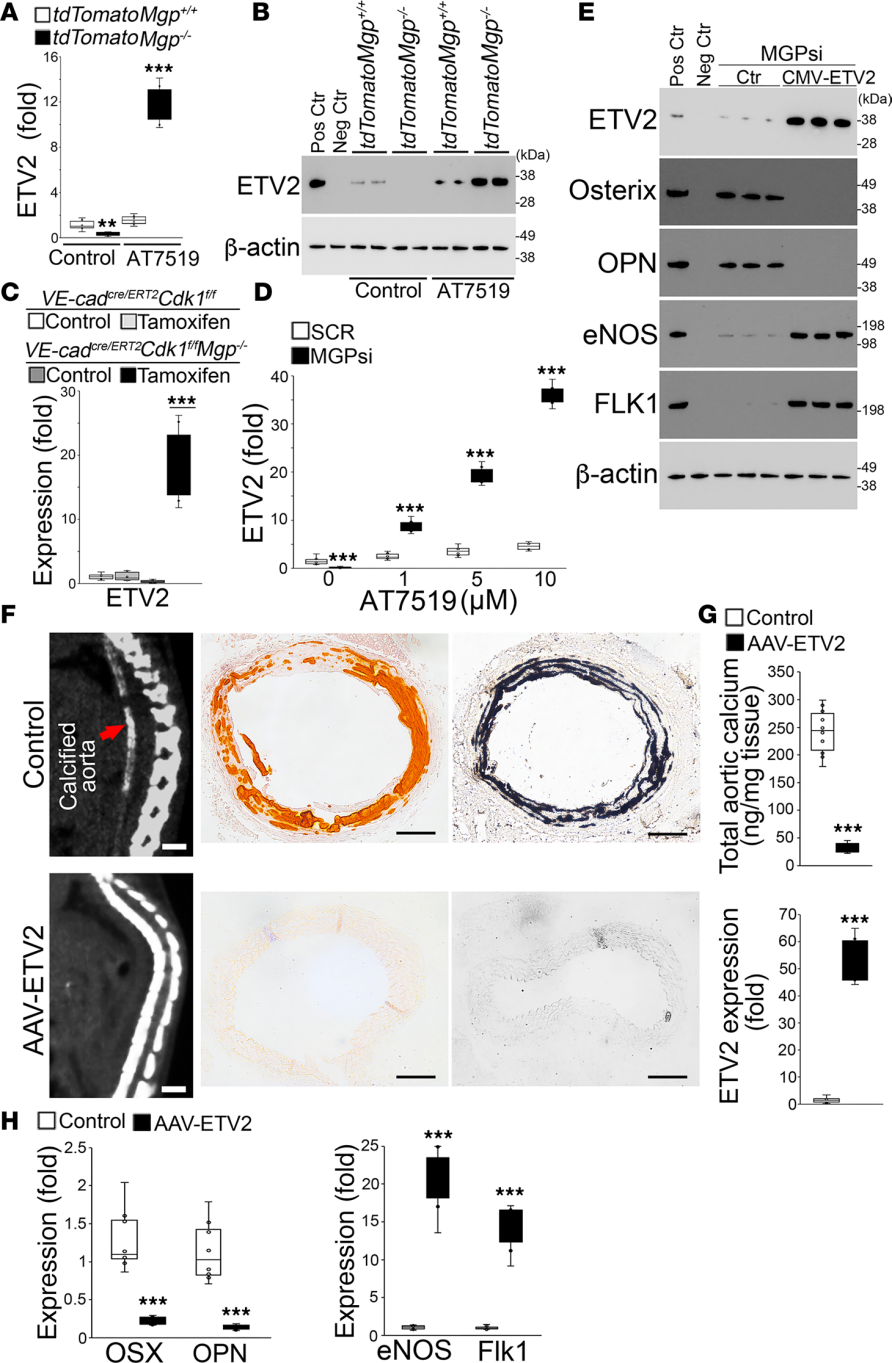
Limiting CDK1 upregulates ETV2 to prevent endothelial lineage cells from undergoing osteogenic differentiation. (**A** and **B**) Expression of ETV2 in tdTomato^+^ aortic cells isolated from *VE-cadherin^cre/ERT2^* *Rosa^tdTomato^* *Mgp^–/–^* mice and *VE-cadherin^cre/ERT2^* *Rosa^tdTomato^* mice, as shown by real-time PCR (**A**) and immunoblotting (**B**) (*n* = 6). Positive control (Pos Ctr) for ETV2 blotting: Mouse ECs induced from embryonic stem cells (66). Negative control (Neg Ctr) for ETV2 blotting: HAECs transfected with ETV2 siRNA. Each lane represents an independent experimental group. (**C**) Expression of ETV2 in CD31^+^CD45^–^ aortic cells isolated from *VE-cadherin^cre/ERT2^* *Cdk1^fl/fl^* *Mgp^–/–^* mice and *VE-cadherin^cre/ERT2^* *Cdk1^fl/fl^* mice after tamoxifen administration (*n* = 6). (**D**) ETV2 expression in HAECs after transfection of MGP siRNA in combination with AT5719 treatment (1–10 μM), as shown by real-time PCR (*n* = 6). (**E**) Immunoblotting of ETV2, osterix, osteopontin (OPN), eNOS, and FLK1 in HAECs after transfection of MGP siRNA in combination with infection of lentiviral vectors containing CMV-driven ETV2 cDNA and treatment with osteogenic induction media. Positive and negative controls are the same as described in **B** and Figure 3. Each lane represents an independent experimental group. (**F** and **G**) Micro-CT imaging and Alizarin red and von Kossa staining (**F**), and total aortic calcium with ETV2 expression (**G**) of descending aortas of *Mgp^–/–^* mice treated with adeno-associated viral (AAV) vectors containing CMV-promoter-driven ETV2 cDNA (*n* = 8). Empty vectors were used as controls. Scale bars: 5 mm (micro-CT) and 100 μm (staining). (**H**) Expression of osteogenic and endothelial markers in CD31^+^CD45^–^ aortic cells from *Mgp^–/–^* mice treated with AAV vectors containing CMV-promoter-driven ETV2 cDNA (*n* = 8). Empty vectors were used as controls. Data were analyzed for statistical significance by 1-way ANOVA with Tukey’s multiple-comparison test (**A**, **C**, **D**, and **H**) or unpaired, 2-tailed Student’s *t* test (**G**). The bounds of the boxes are upper and lower quartiles with data points, the line in the box is the median, and whiskers are maximal and minimal values. ***P < 0.0001.

To explore the mechanism underlying the ability of CDK1 inhibition to block the unwanted osteoblastic differentiation in endothelial lineage cells, we screened the expression of transcription factors involved in endothelial differentiation. Real-time PCR and immunoblotting showed an induction of ETV2 in the tdTomato^+^ aortic cells from *VE-cadherin^cre/ERT2^* *Rosa^tdTomato^* *Mgp^–/–^* mice after AT7519 treatment and a reduction without treatment (Figure 5, A and B). ETV2 induction was also found in the aortic tissues of tamoxifen-treated *VE-cadherin^cre/ERT2^* *Cdk1^fl/fl^* *Mgp^–/–^* mice compared with mice without treatment (Figure 5C). In addition, we found a robust reduction of ETV2 in MGP-depleted HAECs (Figure 5D). When we treated the MGP-depleted HAECs with different doses of AT7519 (1–10 μM), the results showed that AT7519 induced ETV2 in a dose-dependent manner (Figure 5D). When ETV2 was overexpressed in MGP-depleted HAECs, expression of osterix and osteopontin was abolished, whereas that of eNOS and FLK1 was enhanced, as determined by immunoblotting (Figure 5E). The results suggested that CDK1 inhibition upregulated ETV2 to prevent osteogenic differentiation in endothelial lineage cells.

To determine the effect of excess ETV2 on vascular calcification, we retro-orbitally injected *Mgp^–/–^* mice at 2 weeks of age with an adeno-associated viral vector (AAV) containing CMV-promoter driven *ETV2* cDNA. At 4 weeks of age, micro-CT showed that the administration of ETV2 significantly decreased the calcification of *Mgp^–/–^* aortas (Figure 5F). Determination of total aortic calcium and Alizarin red and von Kossa staining all confirmed the decrease in calcification (Figure 5, F and G). Real-time PCR showed induction of ETV2 and endothelial markers with a reduction in osteogenic markers in the *Mgp^–/–^* aortas after treatment (Figure 5, G and H). Together, the results support the notion that CDK1 inhibition induced ETV2 to prevent EC-derived osteogenesis and vascular calcification.

### Limiting CDK1 reduced aortic calcification in diabetic Ins2^Akita/+^ mice.

**Figure 6:**
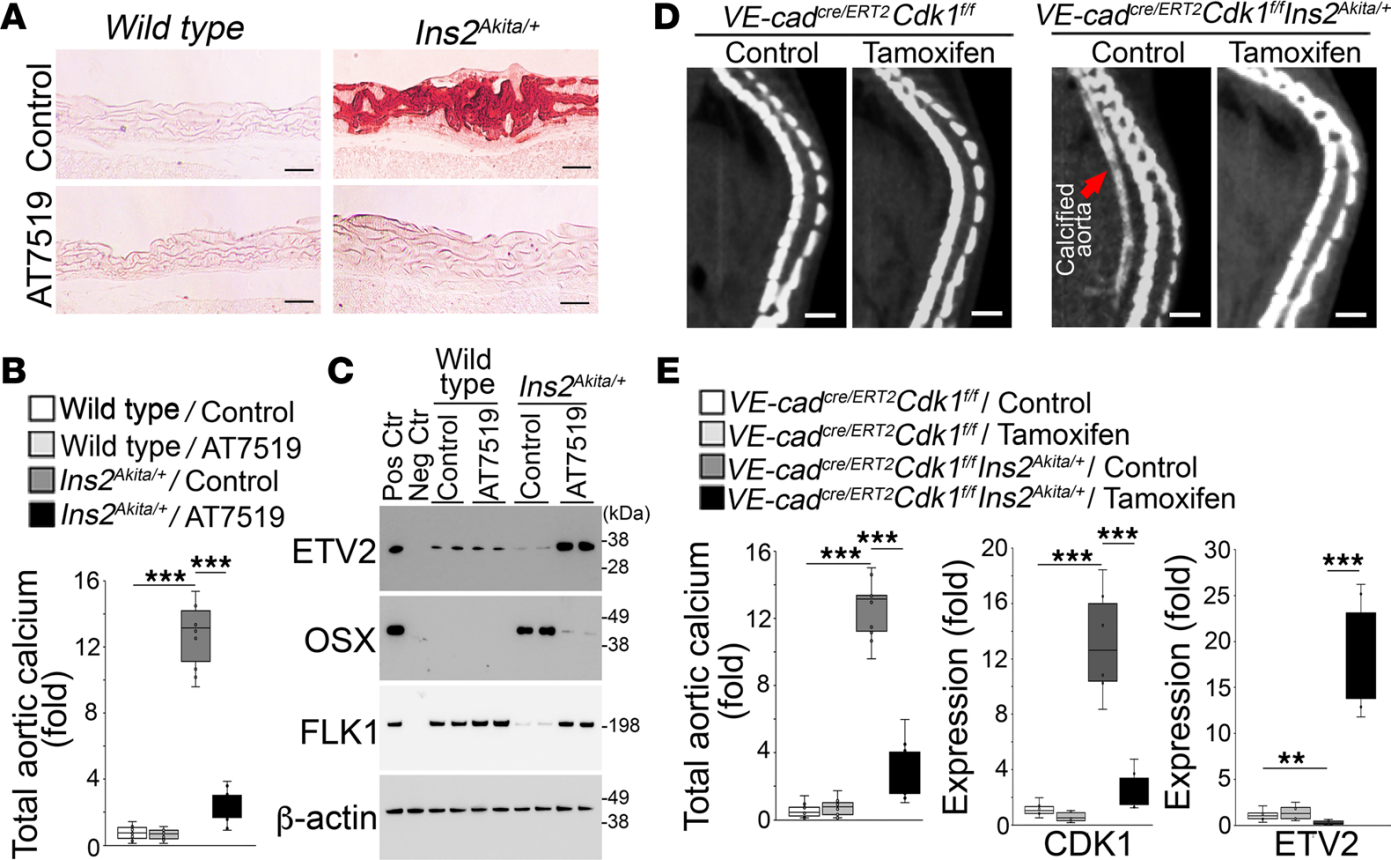
Limiting CDK1 reduces aortic calcification in diabetic *Ins2^Akita/+^* mice. (**A** and **B**) Alizarin red staining (**A**) and total aortic calcium (**B**) of descending aortas of *Ins2^Akita/+^* mice after AT7519 administration (*n* = 10). Scale bars: 100 μm. (**C**) Immunoblotting of ETV2, osterix, and FLK1 in CD31^+^CD45^–^ aortic cells isolated from *Ins2^Akita/+^* mice after AT7519 administration. Positive and negative controls are the same as in Figures 3 and 5. Each lane represents an independent experimental group. (**D**) Micro-CT imaging of descending aortas of *VE-cadherin^cre/ERT2^* *Cdk1^fl/fl^* *Ins2^Akita/+^* mice and *VE-cadherin^cre/ERT2^* *Cdk1^fl/fl^* mice after tamoxifen administration (*n* = 5). Scale bars: 50 μm. (**E**) Total aortic calcium and expression of CDK1 and ETV2 in CD31^+^CD45^–^ aortic cells isolated from *VE-cadherin^cre/ERT2^* *Cdk1^fl/fl^* *Ins2^Akita/+^* mice and *VE-cadherin^cre/ERT2^* *Cdk1^fl/fl^* mice after tamoxifen administration (*n* = 6). Data in **B** and **E** were analyzed for statistical significance by 1-way ANOVA with Tukey’s multiple-comparison test. The bounds of the boxes are upper and lower quartiles with data points, the line in the box is median, and whiskers are maximal and minimal values. **P < 0.001; ***P < 0.0001.

Previous studies have demonstrated that EndMTs drive the endothelium to contribute osteoblast-like cells to the calcifying process in *Mgp^–/–^* mice and diabetic mouse models (14–16, 18, 20). To determine whether limiting CDK1 affected vascular calcification also in diabetes, we used diabetic *Ins2^Akita/+^* mice, in which aortic calcification develops at 30–40 weeks of age (15–18). We initiated AT7519 treatment (5 μg/g, daily) at 34 weeks of age. The *Ins2^Akita/+^* mice were treated for 4 weeks, and saline was used as control. After treatment, Alizarin red staining and total aortic calcium showed reduced mineralization in AT7519-treated mice compared with controls (Figure 6, A and B). Immunoblotting revealed induction of ETV2 and normalization of FLK1 levels but a decrease in osterix in the aortas of AT7519-treated *Ins2^Akita/+^* mice (Figure 6C). The results suggested that limiting CDK1 prevented the EC-derived aortic calcification in *Ins2^Akita/+^* mice.

To determine whether EC-specific deletion of CDK1 improved vascular calcification in diabetic mice, we generated *VE-cadherin^cre/ERT2^* *Cdk1^fl/fl^* *Ins2^Akita/+^* mice. At 10 weeks of age, the mice were injected with tamoxifen (75 mg/kg, daily) for 5 days to delete CDK1 in endothelial lineage cells. *VE-cadherin^cre/ERT2^* *Cdk1^fl/fl^* mice were used as controls. At 40 weeks of age, aortic calcification was determined by micro-CT, which showed a decrease in calcification in the tamoxifen-treated group (Figure 6D). The decreased calcification was confirmed by determination of total aortic calcium (Figure 6E). Real-time PCR showed a reduction in CDK1 with an induction of ETV2 in the aortic tissues of *VE-cadherin^cre/ERT2^* *Cdk1^fl/fl^* *Ins2^Akita/+^* mice after tamoxifen treatment (Figure 6E), suggesting EC-specific deletion of CDK1 decreased vascular calcification in the diabetic mouse model. We also examined the CDK1 expression in different tissues of *Ins2^Akita/+^* mice at 40 weeks of age. Real-time PCR showed induction of CDK1 only in the aortas, but not in bone, lung, brain, kidneys, or liver (Supplemental Figure 1; supplemental material available online with this article; https://doi.org/10.1172/jci.insight.176065DS1), suggesting the specificity of the role of CDK1 in vascular calcification. Together, the results suggested that CDK1 inhibition prevented the EC-derived vascular calcification in diabetic *Ins2^Akita/+^* mice through an induction of ETV2.

## Discussion

The prevention of vascular osteogenesis may have far-reaching public benefits in cardiovascular disease, diabetes mellitus, and kidney disease. In this study, we found that AT7519 treatment or EC-specific deletion of CDK1 limited the generation of EC-derived osteoblast-like cells and decreased vascular calcification through the induction of ETV2. The study provides information for treatment strategies aimed at vascular calcification through the control of ill-fated transitions of endothelial lineage cells. The study also uncovers the cell cycle regulator CDK1 as an important regulator of EC-derived osteogenesis.

The CDK family includes 11 members of classical CDKs (CDK1–CDK11) and 15 other members (29). The CDKs were initially found to play critical roles in cell proliferation by governing the phases of the cell cycle (25). Recent studies also found that the CDKs directly interact with transcription factors that regulate various signaling pathways, such as the p53 or NF-κB pathway (30–34). CDK1 was the first identified member of the CDK family and is essential for cell division in tissue development and regeneration (35–37). In this study, we show that CDK1 is induced in aortic calcification, and EC-specific deletion of CDK1 or an inhibitor of CDK1 AT7519 prevent EC-derived osteogenesis. We detected no induction of other CDKs in EC-derived osteogenesis, suggesting that CDK1 activation is specific to this process. More interestingly, this role of CDK1 may overlap with its function in cell cycle regulation. CDK1 provides key clock regulation for the cell cycle and global gene deletion of CDK1 stops cell division (36–38). The inhibition of CDK1 in the EC-derived osteoblast-like cells might therefore stop the cell clock for their proliferation and the generation of osteoblast-like cells. However, it may also raise concerns that CDK1 inhibition would have off-target effects on other tissues. We examined the CDK1 expression in different organs of *Ins2^Akita/+^* mice and the results showed that CDK1 was only elevated in the aortas (Supplemental Figure 1). Although this result suggests CDK1 inhibition would have the greatest impact on aortic endothelial CDK1, it does not rule out the possibility that CDK1 inhibition would disturb cell proliferation and EC differentiation in other tissues.

ETV2 belongs to the family of ETS transcription factors, which share the ETS DNA binding domain (39). ETV2 plays an essential role in vascular development, angiogenesis, and endothelial reprogramming by directly targeting the genes for endothelial differentiation, including *Flk1, VE-cadherin*, components of the Notch and BMP signaling pathways, and other transcription factors (40–43). Interestingly, the overexpression of ETV2 alone is able to reprogram fibroblasts into endothelial-like cells (44). However, the role of ETV2 in osteoblastic differentiation has never been addressed to the best of our knowledge. In this study, we argue that CDK1 inhibition induces ETV2 to prevent the endothelial transcriptional landscape from shifting toward osteoblastic differentiation, in turn improving vascular calcification. There are several possibilities for endothelial CDK1 to regulate ETV2. CDK1 is a serine/threonine protein kinase and phosphorylates different targets, including proteins of the cell cycle and transcription factors. CDK1 phosphorylates GATA-binding protein 2 (GATA2) and promotes its degradation (45). GATA2 interacts with ETV2 to regulate EC differentiation, and a GATA2 DNA-binding site has been identified in the ETV2 promoter (46). Thus, CDK1 inhibition may cause accumulation of GATA2 and enhance ETV2. CDK1 also phosphorylates the transcriptional activators HCM1, FKH2, and NDD1 and transcriptional repressors YOX1 and YHP1 (47). The CDK1 inhibition could directly alter the activity of these factors to further target ETV2 expression.

ECs contribute to vascular calcification through mesenchymal transitions, which may partly recapitulate a developmental process allowing ECs to regain plasticity. This transition opens a window for unwanted forces to direct EC fate during disease conditions. It also provides the opportunity to control or prevent the transition through the same window. ETV2 is highly expressed in the endothelial lineage during development and is critical for the determination of EC fate (40–43, 46). As we show, if CDK1 inhibition is applied during EndMTs, the elevated ETV2 will prevent ECs moving toward osteogenic differentiation. However, since this abnormal EC-derived osteogenesis is caused by poorly controlled BMP activity, which constantly creates ill-fated ECs, the CDK1 inhibition would have to be continuously applied for ETV2 induction to block the cell transitions completely or partially.

In this study, we focus on the role of ECs in vascular calcification. We show that blocking EC-derived osteogenesis reduces aortic calcification in the *Mgp^–/–^* and diabetic *Ins2^Akita/+^* models. Reports from other investigators have also highlighted other mechanisms leading to calcification in various models. Phosphates and calcium are the key mineral components in calcified vessels and impact the process of calcification. Excess phosphate forces vascular smooth muscle cells to shift toward osteoblastic differentiation, resulting in cellular mineralization (48). In addition, calcium supplementation, but not dietary calcium, has been associated with aortic calcification in postmenopausal women (49). Interestingly, the products of metabolism and biosynthesis have been shown to play roles in vascular calcification. N^ε^-carboxymethyl-lysine, an advanced glycation end product, promotes vascular calcification in a diabetic apolipoprotein E–deficient mouse model through NFATc1 acetylation (50). Advanced glycation end products of bovine serum albumin induce mineralization in smooth muscle cells (51). Total homocysteine in plasma is associated with the severity of infrarenal aortic calcification, possibly through induction of mineralization in mesenchymal stem cells or smooth muscle cells (52). Heparinase, which degrades heparan sulfate, enhances mineralization of smooth muscle cells (53), and N-acetylgalactosaminyltransferase 3 prevents vascular calcification induced by inorganic phosphate through inhibition of the Wnt/β-catenin signaling pathway (54).

Recent studies also reported other pathways in vascular calcification. In diabetic *Ins2^Akita/+^* mice, endothelial expression of sex-determining region Y–box 2 (Sox2) was shown to activate EndMTs and force ECs toward osteoblastic differentiation in response to excess BMP (16–18). In smooth muscle cells, BMP-2 activates msh homeobox-2 (MSX2) to induce mineralization (55), and JNK1 and JNK2 activate c-Jun to regulate extracellular matrix mineralization through directing osteoblastic differentiation (56). Elevated serum levels of platelet-derived growth factor BB promotes vascular calcification in the brain by activating p-PDGFRβ/p-ERK/RUNX2 signaling through crosstalk between pericytes and astrocytes (57). The study also points out that vascular calcification is abnormal ectopic osteogenesis and may not completely follow the normal process of osteogenesis. Therefore, not all osteogenic markers are involved in vascular calcification. For example, increased plasma levels of osteocalcin show no relationship to calcification in chronic kidney disease (58).

Furthermore, recent genetic studies showed a genetic contribution to vascular calcification. Specific single-nucleotide polymorphisms that affect G protein transduction pathways are associated with increased coronary artery calcification (59). As shown by epigenetic studies, histone methylation and acetylation also affect vascular calcification (60, 61). Advanced studies discovered that crosstalk between different types of cells impacts vascular calcification. This includes mesenchymal stromal cells that produce exosomes able to reduce calcification (51). Lymphatic vessel endothelial hyaluronan receptor-1^+^ (LYVE1-1^+^) resident-like macrophages, on the other hand, secrete specific chemokine ligands able to induce mineralization in smooth muscle cells (62). It would be interesting to see whether the mechanism revealed in our study is involved in any of these processes.

In summary, this study targets EC-derived osteogenesis in vascular calcification of *Mgp^–/–^* and *Ins2^Akita/+^* mice and identifies CDK1 inhibition as a potential treatment strategy to reduce vascular calcification.

## Methods

### Sex as a biological variable.

Mixed groups of male and female mice were used in this study.

### Animals.

*Rosa^tdTomato^* [B6;129S6-*Gt(ROSA)26Sor^tm9(CAG-tdTomato)Hze^*/J] mice, *Mgp^+/–^* (B6.129S7-*Mgp^tm1Kry^*/KbosJ), *Cdk1^fl/fl^* [129S(B6N)-*Cdk1^tm1Eddy^*/J] mice, and *Ins2^Akita/+^* (C57BL/6-*Ins2^Akita^*/J) on a C57BL/6J background were purchased from the Jackson Laboratory. *VE-cadherin^cre/ERT2^* mice were obtained as a gift from Ralf Adams (Max Planck Institute for Molecular Biomedicine, Münster, Germany). Genotypes were confirmed by PCR (14, 63) and experiments were performed with generation F4–F6. Littermates were used as wild-type controls. All mice were fed a standard chow diet (Diet 8604, Harlan Teklad Laboratory). Tamoxifen (Sigma-Aldrich, T5648) was injected for 5 days at 75 mg/kg, daily. AT7519 (Selleckchem, S1524) was injected (5 μg/kg, daily) as in previous studies (14).

### Tissue culture.

HAECs (ATCC, PCS-100-011) were cultured as previously described (64). The transfection of MGP siRNA and CDK1 siRNA (Thermo Fisher Scientific, s445791 and 103821) was performed as previously reported (14). AT7519 treatment was performed as described in the main text. Lentiviral vectors containing CMV-ETV2 and AAV vectors containing CMV-ETV2 were all purchased from GeneCopeia and applied to the cells as per the manufacturer’s protocols.

### micro-CT.

Micro-CT imaging was performed at the Crump Imaging Center at UCLA. All the samples were scanned on a high-resolution, volumetric micro-CT scanner (μCT125, built by the Crump Imaging Center at UCLA). The image data were acquired with the following parameters: 10 μm isotropic voxel resolution, 200 ms exposure time, 2,000 views, and 5 frames per view. The micro-CT–generated DICOM files were used to analyze the samples and to create volume renderings of the regions of interest. The raw data files were viewed using the MicroView 3-D volume viewer and analysis tool (GE Healthcare) and AltaViewer software. Additionally, images of the samples were generated using SCIRun (Scientific Computing and Imaging Institute).

### Mouse surgery.

All the surgeries were performed on a heated pad with a connection to a continuous flow of isoflurane. Ectopic bone formation was performed as previously described (28); 5 × 10^5^ cells were mixed with 40 mg hydroxyapatite/tricalcium phosphate powder (Salvin, Orastruct, 0.5 cc) and incubated in a 1 mL syringe at 37°C in 5% CO_2_ overnight. After disinfection with 70% ethanol, a skin incision was made on the back of the mouse. A subcutaneous pouch was formed by blunt dissection. The mixture of cells and hydroxyapatite/tricalcium phosphate was transplanted into the pouch and the incision was closed. The implants were examined by micro-CT imaging and histology 12 weeks after transplantation.

The murine model of hindlimb ischemia was performed as previously described (28). A 10 mm long incision of the skin was made toward the medial thigh. The femoral artery was exposed and separated from the femoral vein and nerve. Silk sutures were used to tie the proximal and distal ends of the femoral artery with double knots. The cells (5 × 10^5^) were transplanted into the surgical area and the incision was closed. Laser Doppler perfusion imaging was used to monitor the blood flow at different time points. Histology and immunostaining were used to examine the vascularization 2 weeks after transplantation.

### RNA analysis.

Real-time PCR analysis was performed as previously described (65). Glyceraldehyde 3-phosphate dehydrogenase (*GAPDH*) was used as a control gene (65). Primers and probes for *MGP*, *Osterix*, *Osteopontin*, *eNOS*, *Flk1*, *CDK1–10*, and *ETV2* were obtained from Applied Biosystems as part of TaqMan Gene Expression Assays.

### FACS.

FACS analysis was performed as previously published (65). The cells were stained with FITC-, phycoerythrin-, or Alexa Fluor 488–conjugated antibodies against osterix (Santa Cruz Biotechnology, sc-22536). Nonspecific fluorochrome- and isotype-matched IgGs (BD Pharmingen) served as controls.

### Immunoblotting and immunofluorescence.

Immunoblotting was performed as described previously (16). Equal amounts of tissue lysates were used for immunoblotting. Blots were incubated with specific antibodies against CDK1 (Thermo Fisher Scientific, MA5-11472), osterix (Santa Cruz Biotechnology, sc-22536), osteopontin, eNOS, and ETV2 (Abcam; ab214050, ab300071, and ab181847), and Flk1 (BD Biosciences, 555307). β-Actin (Sigma-Aldrich, A2228) was used as a loading control. Immunofluorescent labeling was performed as previously described (16) using anti-CDK1. The nuclei were stained with 4′,6-diamidino-2-phenylindole (DAPI; Sigma-Aldrich, D9564).

### Statistics.

The analyses were performed using GraphPad Instat, version 8.0 (GraphPad Software). The distributions of sample values were examined by Shapiro-Wilk normality test using the R function (https://www.r-project.org/) Shapiro.test to confirm that the data in each group were normally distributed. Then, data were analyzed by either unpaired, 2-tailed Student’s *t* test or 1-way ANOVA with Tukey’s multiple-comparison test for statistical significance.

### Study approval.

The studies were reviewed and approved by the Institutional Review Board and conducted in accordance with the animal care guidelines set by UCLA. The investigation conformed to the NIH *Guide for the Care and Use of Laboratory Animals* (National Academies Press, 2011).

### Data availability.

Data are available in the Supporting Data Values XLS file.

## Author contributions

Y Yao and KIB supervised the experiments, analyzed data, and wrote the manuscript. YZ, Y Yang, XW, LZ, XC, JJ, SC, and AV and performed experiments and data analysis.

## Supplementary Material

Supplemental data

Supporting data values

## Version 1 01/23/2024

In-Press Preview

## Version 2 03/08/2024

Electronic publication
