# Supplementary material for: CDK1 inhibition reduces osteogenesis in endothelial cells in vascular calcification
Source: JCI Insight. 2024 Jan 23;9(5):e176065. doi: 10.1172/jci.insight.176065 (PMC10972591; doi:10.1172/jci.insight.176065)
Supplement: Supplemental data [file jciinsight-9-176065-s089.pdf]

CDK1 inhibition prevents osteogenic differentiation in endothelial lineage cells and reduces vascular calcification

Yan Zhao, Ph.D.[1]*, Yang Yang, M.D.[1]*, Xiuju Wu, M.D., Ph.D.[1]*, Li Zhang, Ph.D.[1], Xinjiang Cai, M.D., Ph.D.[1], Jaden Ji, B.S.[1], Sydney Chen, B.S.[1], Abigail Vera, B.S.[1], Kristina I. Boström, M.D., Ph.D.[1, 2, 3], and Yucheng Yao, M.D., Ph.D.[1, 3]

[1] Division of Cardiology, David Geffen School of Medicine at UCLA, Los Angeles, CA 90095-1679, U.S.A.

[2] The Molecular Biology Institute at UCLA, Los Angeles, CA 90095-1570, U.S.A.

*Equal contribution

[3] To whom correspondence should be addressed:

Yucheng Yao M.D. Ph.D. (lead contact) or Kristina I. Boström, M.D., Ph.D.

Division of Cardiology, David Geffen School of Medicine at UCLA

Box 951679, Los Angeles, CA 90095-1679

Tel: 310-825-3239, Fax: 310-206-8553

E-mail: yyao@mednet.ucla.edu or kbostrom@mednet.ucla.edu

Supplemental Figure S1

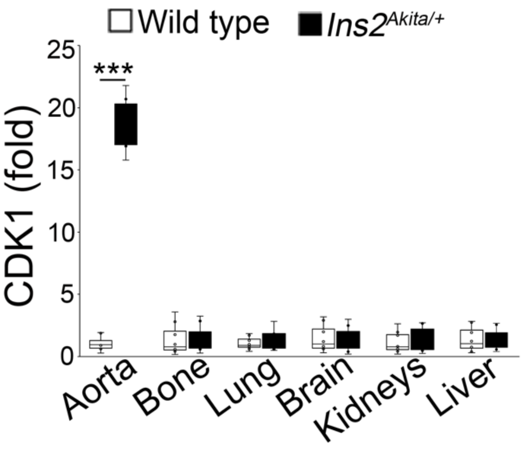

Supplemental Figure S1

CDK1 expression in different tissues of Ins2$^{Akita/+}$ mice shown by real-time PCR (n=8).

Data was analyzed for statistical significance by unpaired 2-tailed Student's t test. The bounds of the boxes are upper and lower quartiles with data points. The line in the box is the median. Error bars are maximal and minimal values. ***, P<0.0001.
